# Metagenomic Analysis Reveals Previously Undescribed Bat Coronavirus Strains in Eswatini

**DOI:** 10.1007/s10393-021-01567-3

**Published:** 2021-12-30

**Authors:** Julie Teresa Shapiro, Sarah Mollerup, Randi Holm Jensen, Jill Katharina Olofsson, Nam-phuong D. Nguyen, Thomas Arn Hansen, Lasse Vinner, Ara Monadjem, Robert A. McCleery, Anders J. Hansen

**Affiliations:** 1 School of Natural Resources and Environment, University of Florida, Gainesville, FL USA; 2 Department of Wildlife Ecology and Conservation, University of Florida, Gainesville, FL USA; 3 University of Lyon, CIRI INSERM U1111 - CNRS UMR5308 - ENS Lyon, 46 Allée d’Italie, 69364 Lyon, France; 4 Centre for GeoGenetics, Natural History Museum of Denmark, University of Copenhagen, Copenhagen, Denmark; 5 Centre for GeoGenetics, GLOBE Institute, University of Copenhagen, Copenhagen, Denmark; 6 Computer Science and Engineering, University of California, San Diego, La Jolla, CA USA; 7 Department of Biological Sciences, University of Eswatini, Private Bag 4 Kwaluseni, Eswatini; 8 Department of Zoology and Entomology, Mammal Research Institute, University of Pretoria, Pretoria, South Africa; 9 Department of Life Sciences, Ben-Gurion University of the Negev, Be’er Sheva, Israel

**Keywords:** Chiroptera, alphacoronavirus, betacoronavirus, emerging infectious diseases, zoonotic disease, human–wildlife interface

## Abstract

We investigated the prevalence of coronaviruses in 44 bats from four families in northeastern Eswatini using high-throughput sequencing of fecal samples. We found evidence of coronaviruses in 18% of the bats. We recovered full or near-full-length genomes from two bat species: *Chaerephon pumilus* and *Afronycteris nana*, as well as additional coronavirus genome fragments from *C. pumilus*, *Epomophorus wahlbergi*, *Mops condylurus*, and *Scotophilus dinganii*. All bats from which we detected coronaviruses were captured leaving buildings or near human settlements, demonstrating the importance of continued surveillance of coronaviruses in bats to better understand the prevalence, diversity, and potential risks for spillover.

**Supplementary Information:**

The online version contains supplementary material available at 10.1007/s10393-021-01567-3.

## Introduction

Coronaviruses are a family of zoonotic viruses comprised of four genera, two of which, alpha- and betacoronaviruses, have an evolutionary origin in bats, while gamma- and deltacoronaviruses, originate in birds (Graham et al. 2013). Coronaviruses have since radiated to a variety of hosts (Drexler et al. 2014). Notably, in humans, coronaviruses have caused COVID-19 (Zhou et al. 2020; Gorbalenya et al. 2020), Severe Acute Respiratory Syndrome (SARS) (Marra et al. 2003; Li et al. 2005), and Middle East Respiratory Syndrome (MERS) (Memish et al. 2013). While recent studies have increased our knowledge of coronavirus diversity and ecology, large gaps in sampling mean there are probably still many undiscovered species and strains in bats (Anthony et al. 2013, 2017).

Southern Africa has a diverse bat community (Monadjem et al. 2020b) that appears to host many coronaviruses, including strains phylogenetically close to MERS-CoV (Geldenhuys et al. 2013, 2018; Ithete et al. 2013), although studies are still limited (Markotter et al. 2020). Globally, the diversity and distribution of coronaviruses in bats makes it likely that future transmission of these pathogens to humans or other animal species will occur (Woo et al. 2009; Anthony et al. 2017). Although there are no known cases of coronavirus spillover in Africa thus far (Markotter et al. 2020), this could occur where bat species come into frequent, close contact with humans or domestic animals (Monadjem 1998; Fenton et al. 2004; Jacobs and Barclay 2009; Noer et al. 2012; Monadjem et al. 2020b).

Therefore, we investigated the prevalence of coronaviruses in bats belonging to eight species from four families (Pteropodidae: *Epomophorus wahlbergi*; Emballonuridae: *Taphozous mauritianus*; Molossidae: *Chaerephon pumilus*, *Mops condylurus*, and *Mops midas*; and Vespertilionidae: *Afronycteris nana*, *Scotophilus dinganii*, and *Scotophilus viridis*). These species are all widely distributed and abundant across southeastern Africa and are commonly found in or near human settlements in northeast Eswatini (Monadjem et al. 2020b, 2021; Shapiro et al. 2020). We subjected fecal samples to virion enrichment followed by RNA sequencing to noninvasively investigate the prevalence and types of coronavirus in the bats of this region. We used this approach to recover whole coronavirus genomes and thus more reliably characterize them (Drexler et al. 2014; De Sabato et al. 2019). This method also allowed us to detect both known and unknown coronaviruses regardless of the specific sequences or genomic region present in samples.

**Figure 1:**
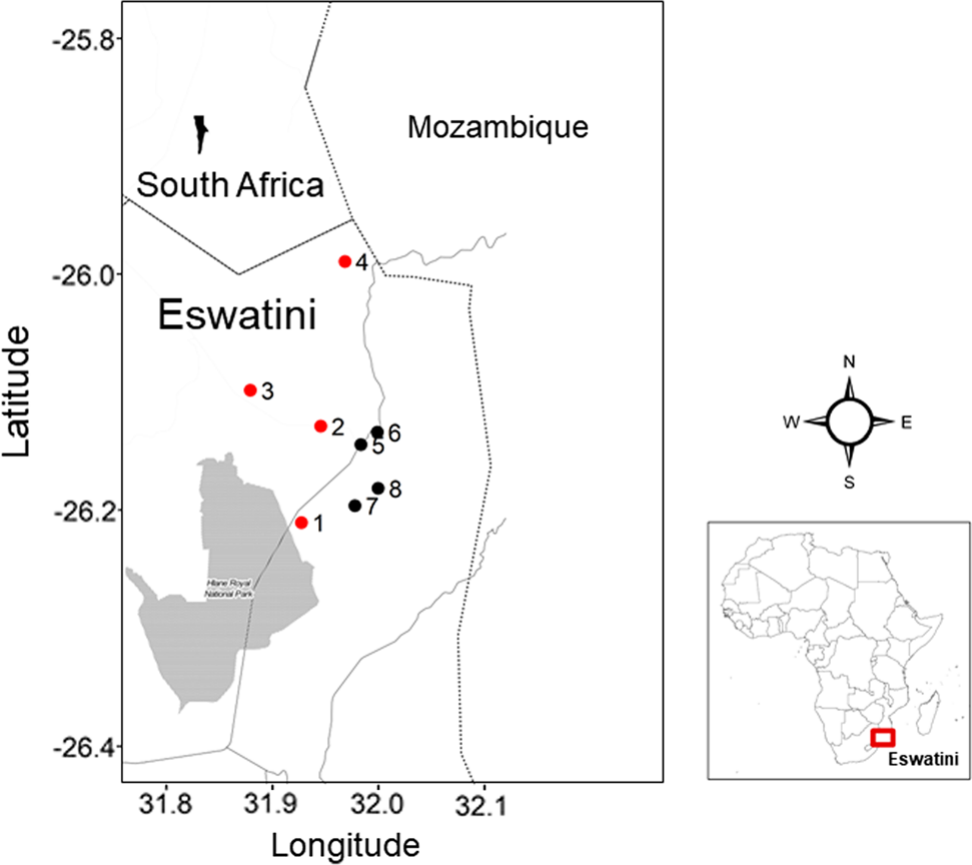
Map of study region. Site numbers indicate where bats were captured and are referenced in Table 2. Sites from which coronaviruses were detected in bats are marked in red, while coronaviruses were not detected in bats captured from sites marked in black. The area shaded in gray is Hlane National Park. Solid lines indicate national borders and dotted lines indicate roads.

We captured bats at eight sites in northeast Eswatini (Fig. 1) from December 2013–May 2014 using mist-nets and/or a harp trap. Taxonomy follows Monadjem et al. (2010, 2020b, 2020a). To aid in the identification of species, we measured forearm length of each captured bat with calipers to the nearest 0.1 mm and mass to the nearest 0.5 g with a spring balance. Captured bats were placed individually in cloth holding bags for the deposition of feces. We trapped, handled, and released bats in accordance with a permit from the Eswatini National Trust Commission and University of Florida Institutional Animal Care and Use Committee approval (Protocol #201,508,751).

Fecal samples from insectivorous species were desiccated and preserved with silica gel (Sigma-Aldrich), while samples from the frugivorous *Epomophorus wahlbergi* were placed in RNALater (Thermo Fisher Scientific) because due to their wet condition they could not be properly dried. Samples were stored at − 10 °C until the end of the field season (May 2014), then transferred to − 80 °C.

Frozen fecal samples were vortexed vigorously in 600 µl of PBS with beads from the PowerFecal kit (MoBio) for 1 min and incubated at room temperature for 10 min. Following incubation, samples were vortexed for 1 min, then centrifuged at 2500 × g for 3 min. The supernatant was then filtered and the flow-through nuclease-treated following Jensen et al. (2015). Viral nucleic acids were subsequently extracted using Roche High Pure Viral RNA kit (Roche) according to the manufacturer’s guidelines after which 1 µl RNase Out (Invitrogen) was added to the final RNA extract (Jensen et al. 2015; Hansen et al. 2015).

Forty-four RNA libraries were produced, each one from an individual fecal sample, using ScriptSeq v2 RNA-seq library preparation kit (Epicentre, Illumina), according to the manufacturer’s guidelines. Samples were DNase-treated with Promega DNase for 30 min at 37 °C and purified on RNeasy MinElute columns (Qiagen). Seven or eight individually and uniquely single-indexed sequencing libraries were pooled together in equimolar ratios for sequencing with paired-end reads of 100 bp (PE100) on an Illumina Hiseq 2000 platform. The library from one sample (Bat50) was resequenced individually on one lane of PE100 on an Illumina Hiseq 2000 platform.

Reads with overlapping sections of sequences were assembled into longer contiguous sequences (contigs) using Ray Meta v2.2.0 with default settings (Boisvert et al. 2012). The contigs were searched for coronaviruses using megablast and BLASTn on the NCBI Nucleotide collection (nt) database (Altschul et al. 1990, 1997) and by mapping against NCBI’s nr database using DIAMOND (Buchfink et al. 2014).

De novo assembly with an alternative assembler was attempted on the eight coronavirus-positive samples using MEGAHIT v1.1.1 (Li et al. 2015) with the following parameters: minimum contig length = 100, minimum kmer size = 15, maximum kmer size = 101, increment of kmer size of each iteration = 2. To search for potential coronavirus genomes, the 20 longest contigs from each assembly were selected and analyzed using BLASTn on the nt/nr databases, which resulted in the identification of longer coronavirus contigs spanning and extending shorter contigs already identified. Further assembly was attempted on the combined set of contigs using Geneious v.11 software (https://www.geneious.com/), resulting in full or near-full genomes for four bats. Reads were mapped back to the genomes using bowtie2 (Langmead and Salzberg 2012) to correct ambiguous bases.

We also mapped all the sequenced reads from individual samples back to the coronavirus contigs using bowtie2 (v2.2.9) (Langmead and Salzberg 2012). We did this in order to confirm which samples the sequences came from and identify any potential cases of bleed over (the misidentification of the sample from which each sequence read originated) following Kircher et al. (2012) and Jensen et al. (2015).

**Figure 2:**
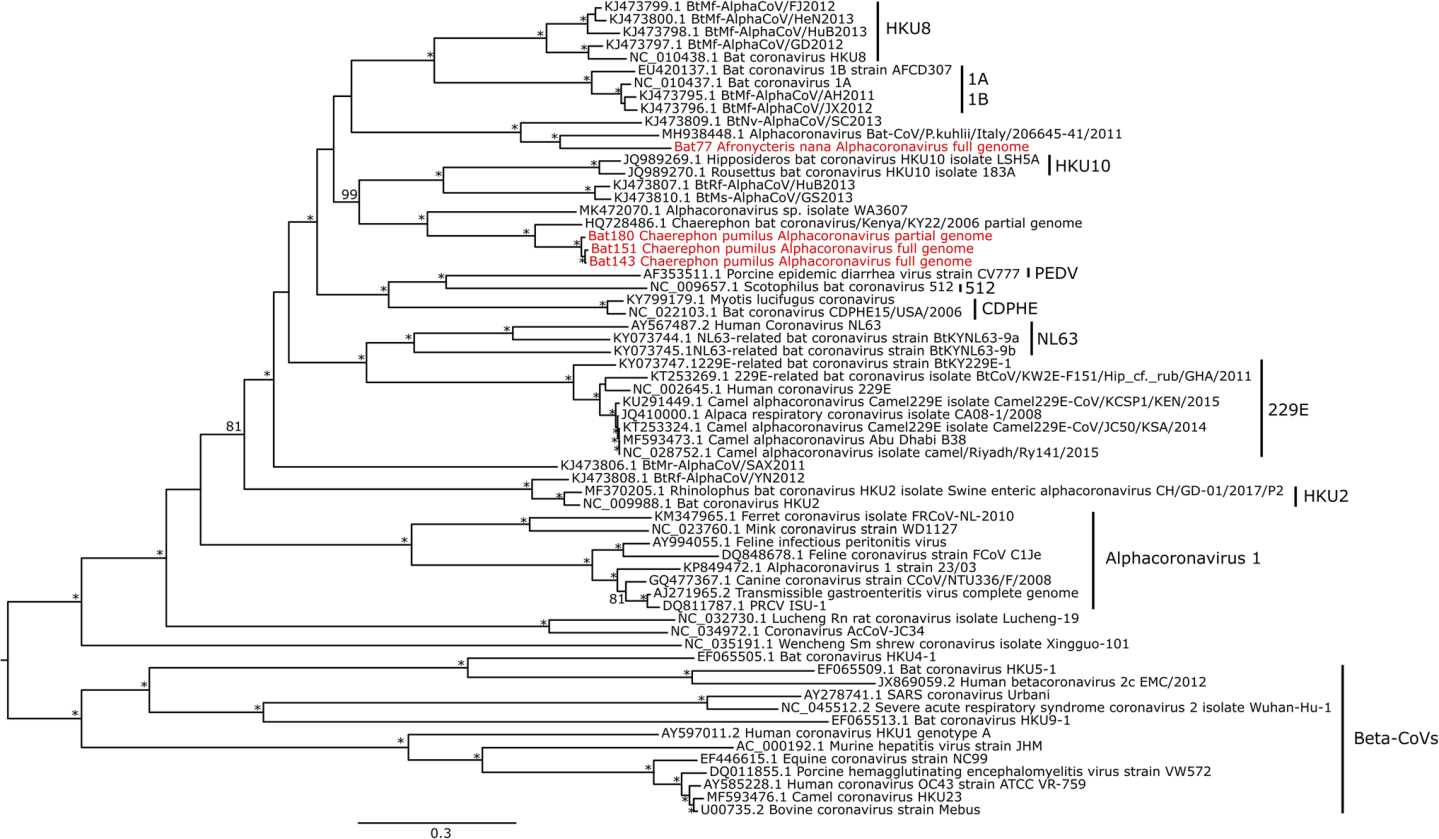
Maximum likelihood phylogeny of coronaviruses (CoVs) based on full genomes, including reference genomes and the four full-length and partial genomes from this study, which are labelled in red and can be retrieved under accession numbers OL807608, OL807609, OL807610, OL807611. Stars indicate branches with 100% bootstrap support.

We identified three full-length and one partial alphacoronavirus genomes from four individual bats (accession numbers OL807608, OL807609, OL807610, OL807611; Supplementary File 1). Three of these were isolated from the species *Chaerephon pumilus*: two of the full genomes (from Bat143 and Bat151; 27,956 nt and 28,061 nt respectively) and one partial genome (Bat180; 20,826 nt). The best hit using BLASTn for all three of these coronavirus genomes was *Chaerephon* bat coronavirus/Kenya/KY22/2006 from Kenya (Tong et al. 2009). When aligned in Geneious, all three were 86–87% identical to this species. Pairwise identity for the ORF1ab gene was 97.1–97.2%, indicating these coronaviruses likely belong to the same species as *Chaerephon* bat coronavirus/Kenya/KY22/2006 based on the coronavirus species demarcation criterion of the International Committee on Taxonomy of Viruses (Lefkowitz et al. 2018; ICTV 2019). In a bootstrapped maximum likelihood tree using RAxML based on full coronavirus genomes following De Sabato et al. (2019), all three *Chaerephon pumilus* coronavirus genomes clustered together as a sister clade to *Chaerephon* bat coronavirus/Kenya/KY22/2006 (Fig. 2). This coronavirus may be widespread within the bat genus *Chaerephon* across Africa. Other coronaviruses have been found in bats of the same species or genera that are geographically distant, sometimes across continents (Drexler et al. 2014) and could indicate connectivity between bat populations across their distribution.

When aligned to each other in Geneious, the three *Chaerephon pumilus* coronavirus genomes were 98.8% identical. The full genomes from Bat143 and Bat151 were slightly more similar to each other (99.4%) than to the partial genome from Bat180 (98.3 – 98.6%). All three genomes were confirmed using real-time PCR using strain-specific primers and fluorescently labeled TaqMan probe designed with Primer3 software in Geneious based on the coronavirus sequences from these three bats (Untergasser et al. 2012) (Supplementary Fig. 1, Supplementary File 2).

We detected a third full-length coronavirus genome from the bat species *Afronycteris nana* (Bat77; 26,977 nt) that likely represents a newly described alphacoronavirus. Its best hit in BLASTn was Alphacoronavirus Bat-CoV/P.kuhlii/Italy/206645-41/2011 isolated from *Pipistrellus kuhli* in Italy (De Sabato et al. 2019). When aligned to Alphacoronavirus Bat-CoV/P.kuhlii/Italy/206645-41/2011 in Geneious, pairwise identity was 76.4% across the full genome and 77.6% across the ORF1ab region. In our phylogeny, this coronavirus was sister to Alphacoronavirus Bat-CoV/P.kuhlii/Italy/206645-41/2011 (Fig. 2). *Afronycteris* and *Pipistrellus* are closely related pipistrelle-like bats in the subfamily Vespertilioninae (Vespertilionidae), albeit in different tribes (Vespertilionini and Pipistrellini, respectively) (Monadjem et al. 2020a); thus it is not unexpected that the coronaviruses from these genera would be relatively similar.

In addition to these full- and near-full-length genomes, we identified 75 shorter coronavirus genome fragments ranging from 103 to 5241 nt from five bats belonging to the species: *Chaerephon pumilus* (Bat180), *Mops condylurus* (Bat166), *Scotophilus dinganii* (Bat167), and *Epomophorus wahlbergi* (Bat50, Bat76) (accession numbers OM000306–OM000380; Supplementary File 3). Only alphacoronavirus sequences were isolated from molossids (*Chaerephon pumilus* and *Mops condylurus*) and vespertilionids (*Scotophilus dinganii*). The sequences detected in the fruit bat *Epomophorus wahlbergi* were either betacoronaviruses (the same genus as SARS-CoV-2 (Zhou et al. 2020; Gorbalenya et al. 2020), SARS-CoV-1 (Li et al. 2005), and MERS-CoV (Memish et al. 2014)), or unclassified coronaviruses (Tables 1, and 2). These sequences were short, ranging from 106–517 nt (mostly < 200 nt), with 71–93% percent identity to previously described coronaviruses. They appear most closely related to coronaviruses sequenced from other pteropodid fruit bats, particularly *Eidolon helvum* in Kenya (Tong et al. 2009), Cameroon (Yinda et al. 2018), and Nigeria (Leopardi et al. 2016), and to a lesser extent *Rousettus leschenaultii* in southern China (Woo et al. 2007). None appeared particularly closely related to any human betacoronavirus pathogens. Targeted PCR of specific genes, such as the RNA-dependent RNA polymerase (RdRp), which is widely used in studies of animal coronaviruses (Drexler et al. 2014), could provide further information about specific lineages of coronaviruses, including the betacoronaviruses. However, lack of material prevents such endeavors at this time.

**Table 1:** Table summarizing coronavirus detection in bats in northeast Eswatini.

Family	Species	Number of captured individuals	Detected CoV (no. of samples)	Proportion of CoV-positive individuals (%)
Pteropodidae	*Epomophorus wahlbergi*	9	2	15
Emballonuridae	*Taphozous mauritianus*	2	0	0
Molossidae	*Chaerephon pumilus*	18	3	17
	*Mops condylurus*	7	1	14
	*Mops midas*	1	0	0
Vespertilionidae	*Afronycteris nana*	1	1	100
	*Scotophilus dinganii*	3	1	33
	*Scotophilus viridis*	3	0	0
Total		44	8	18

**Table 2:** Coronaviruses detected in individual bats.

Family	Species	Bat ID	Capture Site^a^	Number CoV Contigs	Contig length (nt)
Pteropodidae	*Epomophorus wahlbergi*	50	1	36	105–517
		76		6	106–176
Molossidae	*Chaerephon pumilus*	143	3	1	27,956
		151	3	1	28,061
		180	4	6	110–20,826
	*Mops condylurus*	166	4	24	249–4121
Vespertilionidae	*Afronycteris nana*	77	2	1	26,977
	*Scotophilus dinganii*	167	4	4	103–148

^a^Capture site numbers correspond to site numbers Fig. 1.

In conclusion, from a sample of 44 bats in Eswatini, we detected both alpha- and betacoronaviruses. All eight bats from which coronaviruses were detected were captured leaving roosts in houses, churches, or within human settlements. More research is necessary to determine whether any of these detected coronaviruses could be a concern for the health of humans or livestock. Limiting direct contact with these bats or their feces might possibly aid in preventing future emerging infectious diseases, while continued monitoring may shed light on the diversity and ecology of coronaviruses.

## Supplementary Information

Below is the link to the electronic supplementary material.

Supplementary file1 (XLSX 52 KB)

Supplementary file2 (TIF 298 KB)

Supplementary file3 (DOCX 38 KB)
