# Supplementary figures and images for: Metagenomic Analysis Reveals Previously Undescribed Bat Coronavirus Strains in Eswatini
Source: Ecohealth. 2021 Dec 30;18(4):421–8. doi: 10.1007/s10393-021-01567-3 (PMC8718178; doi:10.1007/s10393-021-01567-3)

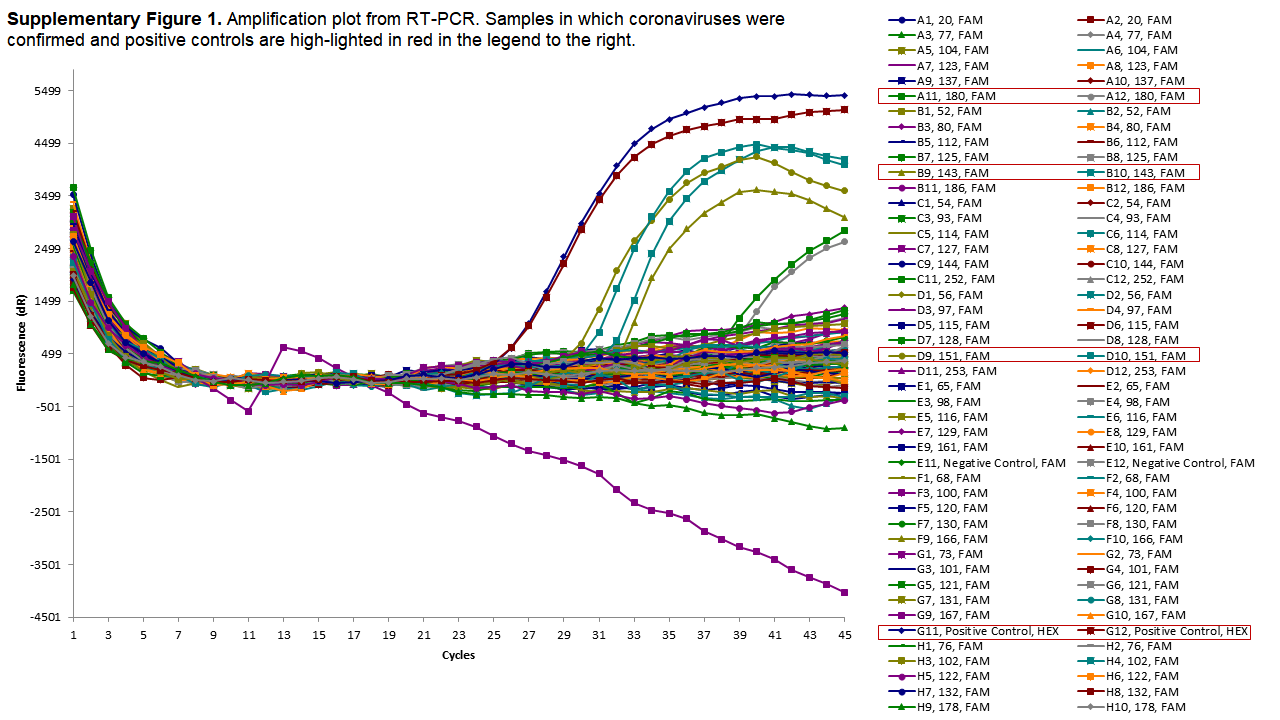

Supplement: Supplementary file 2 — Supplementary file2 (TIF 298 KB) [file 10393_2021_1567_MOESM2_ESM.tif]
