# Supplementary material for: Metagenomic Analysis Reveals Previously Undescribed Bat Coronavirus Strains in Eswatini
Source: Ecohealth. 2021 Dec 30;18(4):421–8. doi: 10.1007/s10393-021-01567-3 (PMC8718178; doi:10.1007/s10393-021-01567-3)
Supplement: Supplementary file 3 — Supplementary file3 (DOCX 38 KB) [file 10393_2021_1567_MOESM3_ESM.docx]

*Response to Reviewers*
**We thank the reviewers for their helpful comments, which have improved our manuscript. We have addressed all their concerns and provide a detailed response to each point that was raised below, in bold print.**

Reviewer: 1

Comments to the Author
In this study, the authors described the discovery of new coronaviruses in African bats using HTS technique. They obtained 3 full and 1 partial genomes that are all alphacoronaviruses but showing variation compared to known ones. Besides, they also found betacoronaviruses in fruit bats, albeit they did not go deep into these viruses. These results add more knowledge of diversity and distribution of bat coronavirus to science. I have two concerns.

**Thank you for your comments, which we have addressed below.**

First, which gene is their phylogenetic analysis based on, ORF1, RdRp or full genome?

**The phylogenetic analysis is based on the full genomes. We have clarified this in the text (page 5): “In a bootstrapped maximum likelihood tree using RAxML based on full coronavirus genomes following De Sabato et al. (2019), all three *Chaerephon pumilus* coronavirus genomes clustered together as a sister clade to *Chaerephon* bat coronavirus/Kenya/KY22/2006 (Figure 2).”**

**We have also added this information to the legend of Figure 2: “Figure 2. Maximum likelihood phylogeny of coronaviruses (CoVs) based on full genomes, including reference genomes and the four full length and partial genomes from this study, which are labelled in red. Stars indicate branches with 100% bootstrap support.”**

Another, the authors should pay more attentions to betacoronavirus, since which includes SARS-related and MERS-related viruses.

**We have added additional information to the betacoronaviruses (page 7): “The sequences detected in the fruit bat *Epomophorus wahlbergi* were either betacoronaviruses (the same genus as SARS-CoV-2 (Zhou et al. 2020; Gorbalenya et al. 2020), SARS (Li et al. 2005), and MERS (Memish et al. 2014)), or unclassified coronaviruses (Table 1, Table 2). These sequences were short, ranging from 106 – 517 bp (mostly < 200 bp), with 71 – 93% percent identity to previously described coronaviruses. They appear most closely related to coronaviruses sequenced from other pteropodid fruit bats, particularly *Eidolon helvum* in Kenya (Tong et al. 2009), Cameroon (Yinda et al. 2018), and Nigeria (Leopardi et al. 2016), and to a lesser extent *Rousettus lechenaulti* in southern China (Woo et al. 2007). None appeared particularly closely related to any human betacoronavirus pathogens.”**

A PCR detection targeting the conserved RdRp can be conducted, then the authors can analyze these amplicons.
**Designing a targeted RdRp PCR to investigate the betacoronaviruses in more details is absolutely possible and we thank the reviewer for this excellent suggestion. However, the fruit bat samples (where we detected the most betacoronaviruses) had very little RNA and we unfortunately had to use up all the material we had for sequencing. It is therefore not possible for us to perform such a targeted PCR. We have added the following text to the manuscript to acknowledge the possibility of doing a targeted PCR to gain more information about the betacoronaviruses (page 7): “Targeted PCR of specific genes, such as the RNA-dependent RNA polymerase (RdRp), which is widely used in studies of animal coronaviruses (Drexler et al. 2014), could provide further information about specific lineages of coronaviruses, including the betacoronaviruses. However, lack of material prevents such endeavors at this time.”**

Reviewer: 2

Comments to the Author
This manuscript describes the metagenomic analysis of fecal samples from a number of species of bats in Africa. This is a timely and relevant topic, highlighting the importance of surveillance of bat populations that are in close contact with humans and domestic animals. A few minor comments below.

**Thank you for your comments. We have responded to each individual comment below.**

1. Introduction: Additional information on the bat species described in the manuscript would be useful such as suborder, species name and geographic distribution.

**We have added this information to the last paragraph of the introduction (page 2-3): “Therefore, we investigated the prevalence of coronaviruses in bats belonging to eight species from four families (Pteropodidae: *Epomophorus wahlbergi*; Emballonuridae: *Taphozous mauritianus*; Molossidae: *Chaerephon pumilus*, *Mops condylurus*, *Mops midas*; and Vespertilionidae: *Afronycteris nana*, *Scotophilus dinganii*, *Scotophilus viridis*). These species are all widely distributed and abundant across southeastern Africa and are commonly found in or near human settlements in northeast Eswatini (Monadjem et al. 2020b; Shapiro et al. 2020; Monadjem et al. 2021).”**

2. Page 2, first paragraph: Please provide additional context about coronaviruses from bats and birds (ie. Bats carry alpha and betacoronaviruses while birds carry gamma and delta coronaviruses).

**We have made this change (page 2): “Coronaviruses are a family of zoonotic viruses comprised of four genera, two of which, alpha- and betacoronaviruses, have an evolutionary origin in bats, while gamma- and deltacoronaviruses, originate in birds (Graham et al. 2013). Coronaviruses have since radiated to a variety of hosts (Drexler et al. 2014).”**

3. p3, line 54: describe minor modifications

**This phrase was an error, library building followed the manufacturer’s instructions without modifications. We have changed the manuscript accordingly.**

4. p4: provide references for programs (Megahit, Geneous and bowtie at first description).

**We have added citations for Megahit (Li et al. 2015) and bowtie (Langmead and Salzberg 2012). According to the website for Geneious, the correct way to cite the software is by the website and therefore we cite it this way in the text.**

5. p6. second paragraph: provide additional information on whether the Pipistrellus bat that is the host to the most closely related CoV is closely related to any of the bats in this study

**We have provided additional information as requested (p. 6): “Both *Afronycteris* and *Pipistrellus* belong to Vespertilionidae but within this family, these genera are found in different tribes (Vespertilionini and Pipistrellini, respectively) (Monadjem et al. 2020a); thus it is not unexpected that the coronaviruses from these genera would be relatively similar.”**

6. p6. third paragraph: there is very little information on the betacoronaviruses despite these being perhaps the most interesting. How similar are these sequences to other betacoronaviruses?

**We have added information on those betacoronaviruses as requested (page 7): “The sequences detected in the fruit bat *Epomophorus wahlbergi* were either betacoronaviruses (the same genus as SARS-CoV-2 (Zhou et al. 2020; Gorbalenya et al. 2020), SARS (Li et al. 2005), and MERS (Memish et al. 2014)), or unclassified coronaviruses (Table 1, Table 2). These sequences were short, ranging from 106 – 517 bp (mostly < 200 bp), with 71 – 93% percent identity to previously described coronavirus sequences. They appear most closely related to coronaviruses sequenced from other pteropodid fruit bats, particularly *Eidolon helvum* in Kenya (Tong et al. 2009), Cameroon (Yinda et al. 2018), and Nigeria (Leopardi et al. 2016), and to a lesser extent *Rousettus lechenaulti* in southern China (Woo et al. 2007). None appeared particularly closely related to any human betacoronavirus pathogens.”**

7. The phylogenetic tree was very difficult to read as were the supplementary tables

**To improve readability, we have submitted the supplementary file as an Excel spreadsheet, which allows readers to scroll, zoom, or adjust font sizes more easily than with the original pdf file. Unfortunately, due to restrictions on file size in the submission portal, we were unable to increase the size or resolution of the phylogenetic tree.**
